# An Atlas of annotations of Hydra vulgaris transcriptome

**DOI:** 10.1186/s12859-016-1172-9

**Published:** 2016-09-22

**Authors:** Daniela Evangelista, Kumar Parijat Tripathi, Mario Rosario Guarracino

**Affiliations:** Laboratory for Genomics, Transcriptomics and Proteomics (LAB-GTP), High Performance Computing and Networking Institute (ICAR), National Research Council of Italy (CNR) Napoli, Italy, Via Pietro Castellino, Naples, 111 Italy

**Keywords:** *Hydra vulgaris*, Annotations, Transcriptome, Database, PHP, MySQL

## Abstract

**Background:**

RNA sequencing takes advantage of the Next Generation Sequencing (NGS) technologies for analyzing RNA transcript counts with an excellent accuracy. Trying to interpret this huge amount of data in biological information is still a key issue, reason for which the creation of web-resources useful for their analysis is highly desiderable.

**Results:**

Starting from a previous work, Transcriptator, we present the *Atlas* of *Hydra’s vulgaris*, an extensible web tool in which its complete transcriptome is annotated. In order to provide to the users an advantageous resource that include the whole functional annotated transcriptome of *Hydra vulgaris* water polyp, we implemented the *Atlas* web-tool contains 31.988 accesible and downloadable transcripts of this non-reference model organism.

**Conclusion:**

*Atlas*, as a freely available resource, can be considered a valuable tool to rapidly retrieve functional annotation for transcripts differentially expressed in *Hydra vulgaris* exposed to the distinct experimental treatments.

**Web resource URL:**

http://www-labgtp.na.icar.cnr.it/Atlas.

## Background

*Hydra vulgaris* is a small fresh water organism belonging to genus Hydra of the phylum cnidaria and class hydrozoa. The genus Hydra is well known for its regeneration capability, firstly observed by Abraham Trembley in 1744. Since the last two hundred years, it attracts the interest of the scientific community because of its unique regeneration ability, and it appears not to age or die’ status. In particular, researchers show interest in studying Hydra as model organism with respect to diverse biological research realms ranging from embryogenesis [1], nervous system development [2], aging mechanism [3], and to the effects of toxicity in ecosystems [4]. Recently, hydra also become very popular in stem cell research due to the inherent nature of its specific ectodermal, endodermal epithelial and interstitial stem cells [5]. Though the cellular organization of hydra is well established, researchers are working on the molecular mechanisms behind the above mentioned aspects of hydra, more specifically at the molecular level. In 2010, a draft genome of *Hydra magnipappilata* [6] was reported. Recently, the transcriptomics analysis of hydra [7] has been carried out to unveil the genetic cascades upholding the biological demeanor with respect to regeneration ability, such as immunity, cell cycle regulation, cell death, transcription and chromatin regulation. However, generally in case of Hydra, the interpretation of transcriptomics data in the absence of well annotated genome or transcriptome is a difficult task, and without the help of biologists friendly tools, it appears to be a problematic case. By searching the literature, we observed that only two web resources are available: Compagen [8] and Cnidbase [9]. On the one hand, Compagen basically stores all the raw and processed sequences from sponges, cnidarians, tunicates and lower vertibrates to retrospect evolutionary relationship among them. It is a comparative genomics platform, though it lacks in reflecting any functional annotation aspect of sequences associated to hydra genus. On the other hand, Cnidbase is a evolutionary genomics database, which basically highlights the evolutionary relationship among various species in phylum Cnidaria. Both these resources does not provide functional aspects of the Hydra transcripts. To acknowledge this limitation, we previously developed a HvDbase database to integrate 15,522 transcripts along with their functional information [10]. We upgraded this resource to develop a new web application *Atlas* to store *Hydra vulgaris* specific transcripts and annotate all the relevant functional information with respect to GO terms, pathways, protein domains and other important data and information using Transcriptator software [11]. *Atlas* is an easy to use application to obtain functionally related information for each and every transcript. Each entry is also hyper-linked with external database for crosschecking and further downstream analysis. At present, around 70 % of the *Hydra vulgaris* transcritome is annotated and managed by Atlas application.

## Methods

### Transcriptomic data retrieval

The *Hydra vulgaris* RNA-Seq transcriptomic data were published in a prevoius research work [7]. They produced RNA-Seq transcriptome by Illumina and 454 reads obtained from the*Hydra vulgaris* strain "Basel". The assembly of reads were carried out by both genome assisted (using *Hydra magnipapillata* genome) as well as de-novo based assembly. Finally, a dataset was obtained with the longest ORFs, both from genome assisted and de-novo assembly methodology was obtained. It contains 48,909 sequences, out of which the 45,269 transcripts longer than 200 base pairs have been deposited to European Nucleotide Archieve (ENA), with accession numbers HAAC01000001-HAAC01045269. We retrieved the raw transcripts data for annotation purposes and carried out our downstream analysis.

### Database content

Atlas web application is designed to accommodate a vast amount of information ranging from Gene Ontological (GO) terms related to biological activity, molecular function and cellular components with respect to each stored transcript. It also took into account associated protein domain information from various protein domains databases such as COG, Inter-Pro, PFAM and SMART. In *Atlas*, we also include enriched pathways information from KEGG, Panther, BioCarta for each given transcripts related to *Hydra vulgaris*. This information is relevant to dissect high level biological function and biomolecular interaction network in cellular context. It also provides information about interaction partners for the protein products of the respective transcripts. To gather this information, protein interaction databases BIND and MINT are exhaustively searched and indexed in *Atlas*. Apart from functional aspects, it also reports other relevant information from Swiss-Prot, UniProt-Knowledgebase and OMIM.

### Pipeline

*Atlas* application is based on Transcriptator workflow [11, 12]. This pipeline employs web-services from DAVID [13, 14] and Quick-GO [15]. DAVID web-service client is written in python utilizing light weight soap client suds-0.4 module [16]. The client for Quick-GO uses python package ’Bio-Services’ and provides wrapper framework based on wsdl/SOAP and REST protocols to the basic pipeline. The main purpose of the pipeline is to annotate the given transcript(s) for functional and biological relevant information. To achieve this, it carries out the processing in four main steps: a) finding the best hit protein for a given transcript sequence in locally installed Swiss-Prot [17] and Uni-Prot [18] Blast [19] formatted databases; b) obtaining functionally relevant information for best hit protein from DAVID database; c) assigning GO slim terms to these protein hits from Quick-Go database; d) integrating all the relevant information in tabular and graphical format for the respective best hit protein, for the given transcript. Blast search is carried out on local cluster, while the second and third steps simultaneously employs the above mentioned DAVID and QUICK-GO web-services. The last step, integrates the results and carry out statistical analysis and generate easy to read tables and graphical charts.

### Application framework

**Fig. 1:**
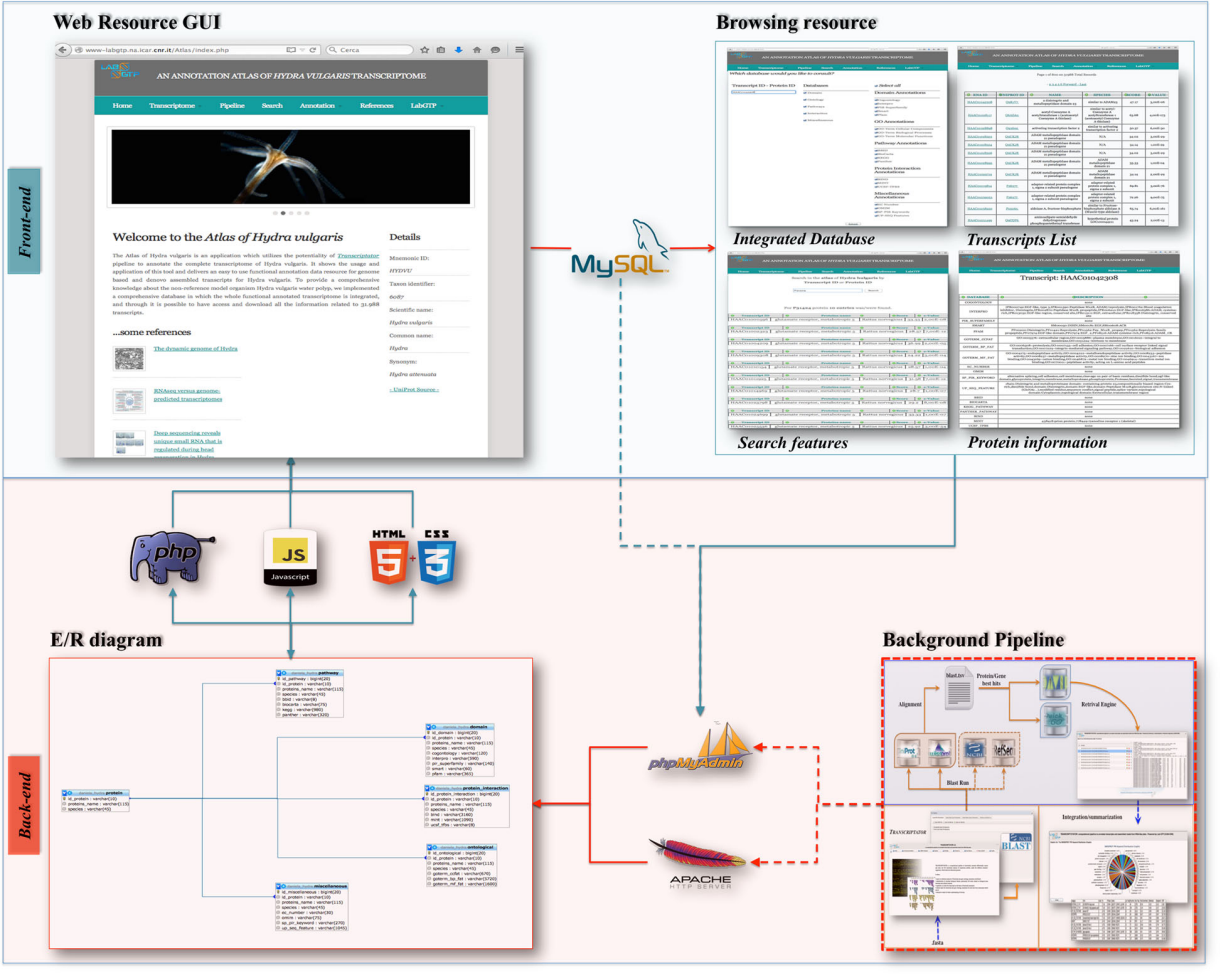
The bioinformatics pipeline of the *Atlas* of Hydra vulgaris

We have developed the database and a web resource to extract and display all the collected contents, some of which derive from external repositories. Indeed, Atlas is designed to be an integrated system with the principles of a web orientated architecture (WOA). By quering the background relational database, it matches data with common features found within the dataset and it returns them in tables specifically structured for providing a comprehensive and well-organized visualization. In detail, *Atlas* planning is based on an Entity–Relationship diagram which describes interrelated characteristics of gathered information (Fig. 1). The proposed back-office system is shown in the lower rectangle, while the front-office is represented in the upper one. The connection between the two is obtained through an abstraction layer, which enables the modularity and future extension and upgrade of the system. The implemented home page (upper left corner) and the available web-sections (upper right corner) are presented in a comprehensible and easy to read way to help scientist in searching, visualizing and downloading the data.

### Resource development and description

Atlas application, as the previous version [10], is a database-driven web site, based on a Relational Database Management System. The back-office’s structure and the GUI’s design are developed using de facto standard technologies both in scientific and commercial environments [20]. Indeed, Atlas works on a web server Apache/2.2.26 [21]; MySQL client version 5.3.28 - 10.04.1 (Ubuntu) [22] and the freely available tool phpMyAdmin version 3.3.2 deb1 Ubuntu 0.2 [23] useful for remote MySQL administration. The dynamic contents of the front-end have been implemented in PHP/5.2.6-3 [24] and JavaScript [25]; HTML5 [26] and CSS 3.0 [27] are used for static contents (Fig. 1). The Markup Validation Service (MVS) of the World Wide Web Consortium (W3C) [28] was used for code approval. The web application that although optimized for Safari, runs on all browsers and it is also reachable from smartphones. The application provides the opportunity to retrieve data, such as: transcriptID or proteinID, in three different ways (Fig. 4). First, from the drop down menu, by selecting the Transcripts list section, users are able to visualize the complete list of transcripts and their annotation can be retrieved. From there the user will be redirected to the functional annotation page. Then, by using the Database list section, user can have the option to select a specific type (transcript or protein ID), as well as the categories of annotation of interest and be redirected to the integrated table page. Finally, the *Search* section, provides the opportunity to insert a specific transcript to acquire the related information, for example if a user has a protein ID, it will be possible to obtain the list of all available associated transcripts of *Hydra* and viceversa.

## Results and discussion

### General framework of *Atlas*

*Atlas* consists of seven sections, among which the *Transcriptome* section, conceived to contain two separate web pages *Transcripts List* and *Database List*, represents the resource’s core. The *Transcript List* subsection hosts the whole transcripts list of the *Hydra vulgaris* transcriptome, as well as associated functional annotations through custom made Python scripts to access open source tools and public databases (Fig. 1). The second subsection *Database List* queries the five database sets, which we have hosted and suitably merged in: Domain, Ontology, Pathways, Interaction and Miscellaneous. All the other sections were considered to host in-depth pages contents of the web application.

### Data organization

*Atlas* collects data for 19 different functional terms, deriving from scientific repositories and integrates them in tables that can be ordered by column and filtered for features, in order to be easily readable. The information of each single transcript were organized, under Transcripts List web-page, in: Ena Id, Uniprot Id, Name, Score, E-Value and, under Databases List web-page, in five databases groups in which additional specific descriptions are reported. Moreover, all parameters or databases have more in-depth explanations at the bottom of the page.

### Statistical analysis of *Hydra vulgaris* transcripts

**Fig. 2:**
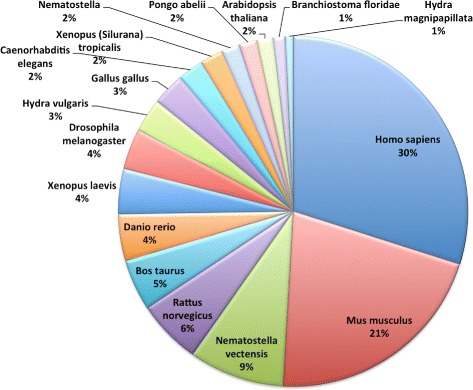
Species distribution

**Fig. 3:**
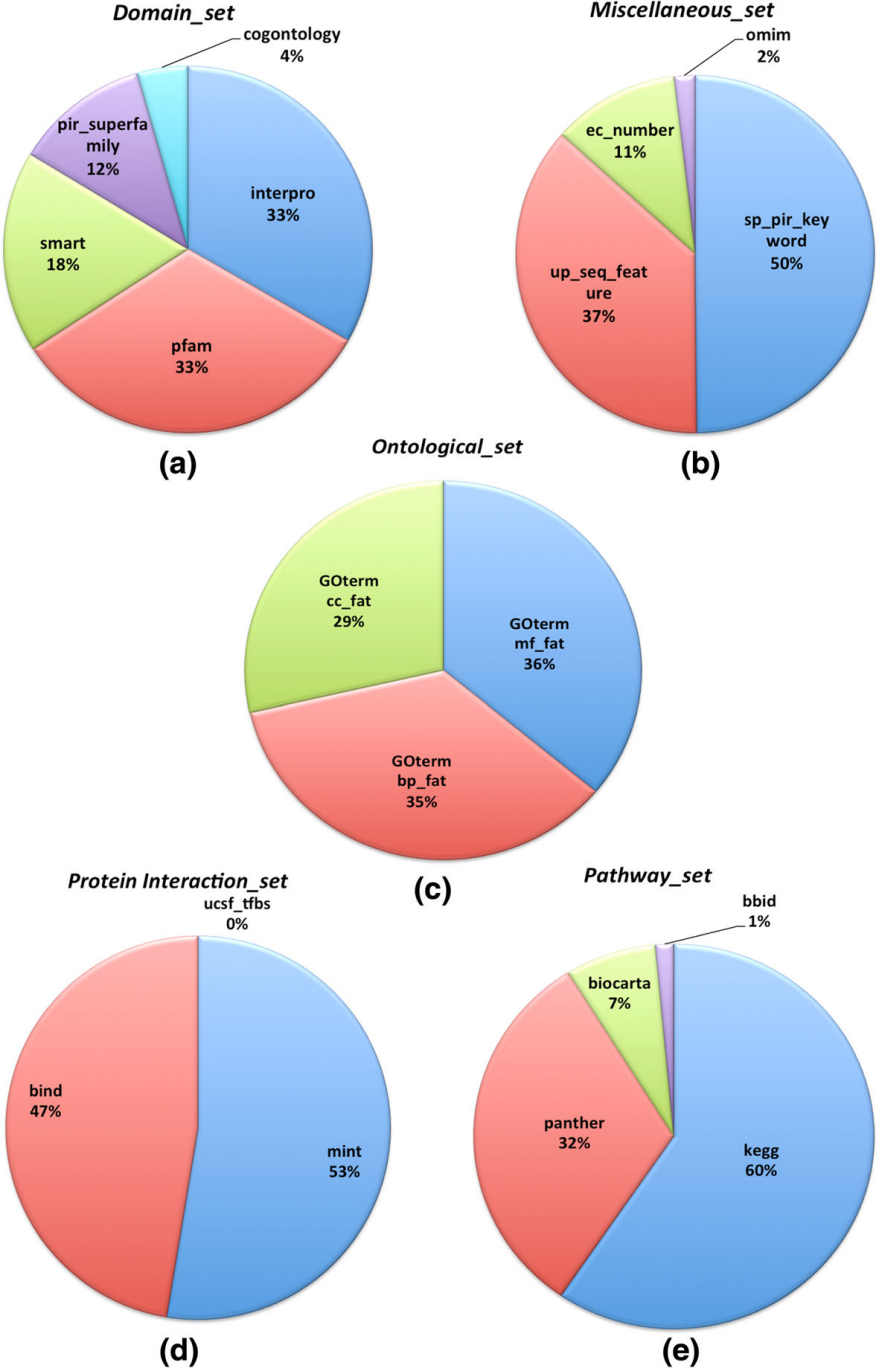
Functional annotation distribution of *Hydra vulgaris* transcriptome in the *Atlas*. **a** Domain annotation category shows Interpro and PFAM as the more highly enriched domains. **b** Miscellaneous category shows that SP-PIR keywords annotation occupies the half of the terms enriched within the databases. **c** Gene Ontology category shows that biological processes (BP) and molecular functions (MF) share the similar level of enrichment. **d** Protein interaction category shows that Mint and Bind databases are almost equally distributed. **e** Pathway category shows that KEGG and Panther are the most over expressed terms in relation with the stored transcripts

To carry out functional and gene ontology annotation for the obtained proteins, the DAVID (Database for Annotation, Visualization and Integrated Discovery) [29, 30] web resource has been used. Out of 31.988 protein hits, only 60 % (18,133) protein ids are annotated with the help of the DAVID web resource (Table 1), whereas, the annotations for the remaining protein ids were not present in it. Moreover, in *Atlas*, various types of annotation details with respect to each matched protein (corresponding to *Hydra vulgaris* transcripts) are stored. To showcase the enrichment of these functional and gene ontology categories, with respect to the total population of available transcripts in *Atlas*, distribution plots for various functional and GO-terms categories are provided. In Fig. 2, species distribution represents the top seventeen species, for which the BlastX program obtained the significant proteins hits with maximum score for the *Hydra vulgaris* transcripts. It is evident that most of the proteins hits belong to reference models (*Homo sapiens* as well as *Mus musculus* (20-30 %)) which are very well annnotated in the Swiss-Prot and UniProt-trEMBL databases. In a similar way, *Atlas* contains several biological and functional annotation categories in relation to the 18,133 proteins ids (60 % of *Hydra vulgaris* transcriptome). Domain annotation category (Fig. 3a) shows Interpro (33 %) and PFAM domains (33 %) are highly enriched in the *Hydra vulgaris* transcriptome. Gene Ontology categories (Fig. 3c) such as, biological processes (BP) and molecular functions (MF) share the similar level of enrichment (35 %), while cellular components (CC) (29 %) are represented by fewer transcripts. For miscellaneous and protein interaction annotation terms, SP-PIR keywords annotation (50 %) in miscellaneous category (Fig. 3b) and BIND (47 %)/MINT (53 %) in protein interaction databases (Fig. 3d) draw attention to higher distribution among the *Hydra vulgaris* transcriptome. The Pathway statistics (Fig. 3e) shows that KEGG (60 %) and Panther (32 %) are the most prominent pathways terms associated with the transcripts stored in the *Atlas*. The rest of functional terms in these two categories share a smaller coverage of transcripts from 0–40 % (Fig. 3b and d).

**Table 1:** The *Atlas* content at a glance

	ENA	BlastX Hits	Proteins obtained	DAVID annotation
Trans >200	45.269	31.988	18.133	13.761
Trans <200	3.640	n/a	n/a	n/a
LeftOver	n/a	13.281	n/a	4.372
TOT	48.909	45.269	18.133	18.133

### A case study

**Fig. 4:**
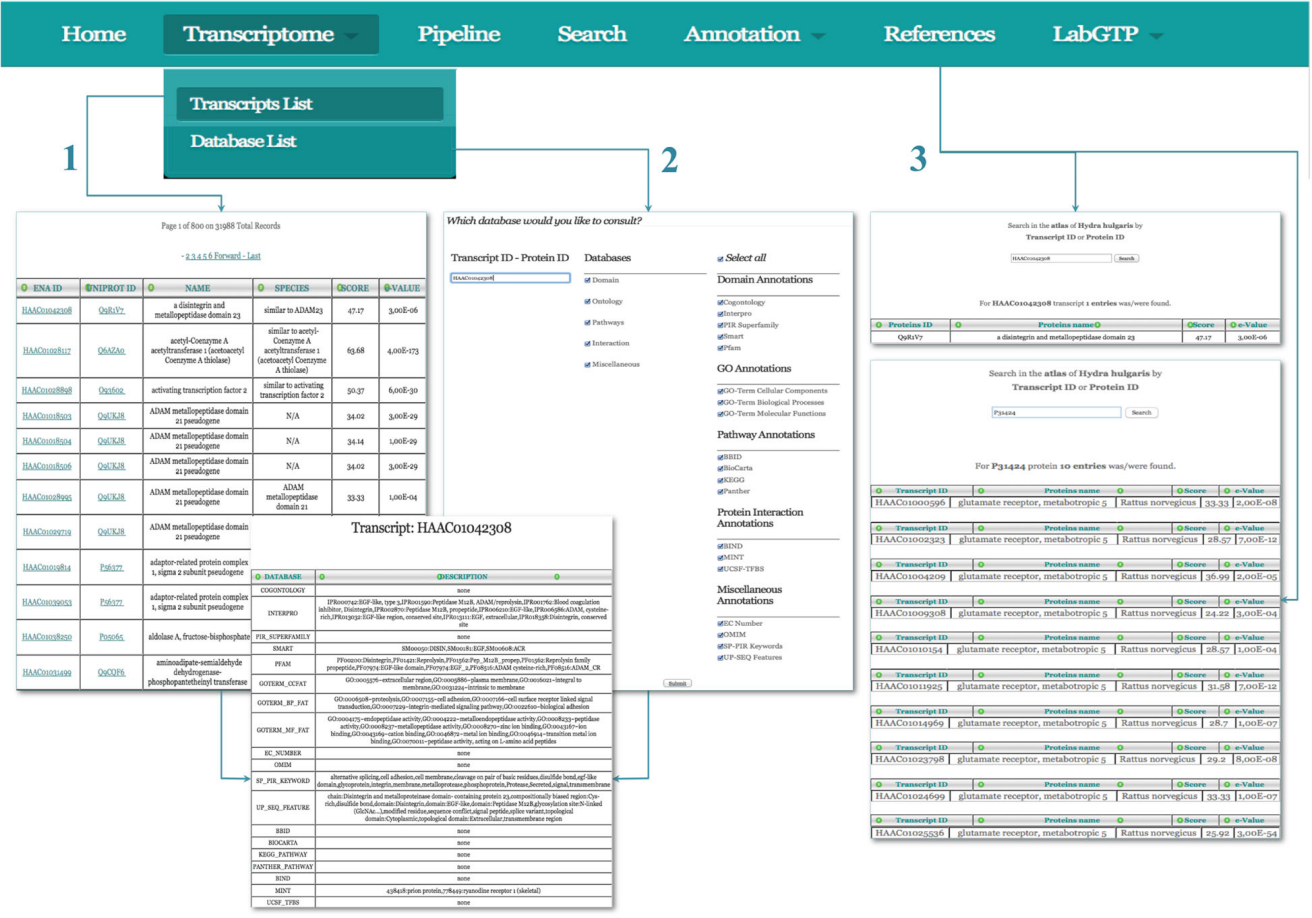
Data retrieval path of the HAAC10000596 transcript

We present, in Fig. 4, a case study for the retrieval of the functional annotation information for the HAAC01042308 transcript. By running Blastx (comparative genomics approach), we obtain Q9R1V7 protein id from uni-prot as best hit. GO annotation obtained for this protein hit suggests its most possible biological role in cell adhesion, cell surface receptor linked signal transduction. The molecular function associated to it refers to metalloendopeptidase activity and cellular location is confined to plasma membrane. The domain which are associated with this protein hist is ADAM/reprolysin domain. By combing all these information from different resources, it is possible to suggest that the possible product of this transcript is metalloprotease-like protein engage in inter-cellular interaction as well cellular interaction with the extra-cellular matrix. While cross checking the best hit protein (hyper link is provided for each hit) for the given transcript in uniProt Knowledge base, we observe that the protein is Disintegrin, and metalloproteinase domain-containing protein 23 and product of Gene ADAM 23 reported in *Mus musculus*. Cross checking the result with the functional annotation obtained from Atlas application, it is possible to describe the putative biological role of this unknown transcripts with in *Hydra vulgaris*. Similarly, obtaining functional annotation details using and cross checking with external database, enables Atlas application to characterize unknown transcripts of *Hydra vulgaris*, generated through different transcriptomic experiment in a simple way.

## Conclusion

The new high-throughput technologies allow us to sequence new organisms in a fast and easy way, but the problem they pose is to infer the relevant information in the huge amount of data returned from the experiments. A database designing devoted to non-reference model organisms is needed. We have developed an elegant approach to address the de-novo assembled reads from *Hydra vulgaris* and to formulate the structure to handle the functional annotation information for all those organisms which are not referenced, and for which there is very little information. *Atlas* is an intuitive and easy-to-use web resource for researchers interested in studies of this non-reference model organism which can be extended to the cases where the transcriptome is available, but the genome is not yet well annotated. *Atlas* has been designed to integrate 19 repositories of functional annotations and several functionalities, for which it is possible to gain access without credentials. Moreover, being a modular platform, it is easily scalable and customizable for future demands and developments. This work is likely to constitute an interesting starting point for developing similar web-resources. Indeed, we are processing new functional annotation data, in order to upgrade the *Atlas* and make it much more informative and attractive.
